# Potassium usnate, a water-soluble usnic acid salt, shows enhanced bioavailability and inhibits invasion and metastasis in colorectal cancer

**DOI:** 10.1038/s41598-018-34709-9

**Published:** 2018-11-02

**Authors:** Yi Yang, Woo Kyun Bae, Ji-Yoon Lee, Yong Jae Choi, Kyung Hwa Lee, Myong-Suk Park, Young Hyun Yu, So-Yeon Park, Rui Zhou, İsa Taş, Chathurika Gamage, Man-Jeong Paik, Jae Hyuk Lee, Ik Joo Chung, Kyung Keun Kim, Jae-Seoun Hur, Sang Kyum Kim, Hyung-Ho Ha, Hangun Kim

**Affiliations:** 1 College of Pharmacy and Research Institute of Life and Pharmaceutical Sciences, Sunchon National University, Sunchon, Korea; 2 Korean Lichen Research Institute, Sunchon National University, Sunchon, Korea; 3 Department of Hematology-Oncology, Chonnam National University Medical School, Gwangju, Korea; 4 College of Pharmacy, Chungnam National University, Daejeon, Korea; 5 Department of Pathology, Chonnam National University Medical School, Gwangju, Korea; 6 Department of Pharmacology, Chonnam National University Medical School, Gwangju, Korea

## Abstract

Usnic acid (UA), a lichen secondary substance, has considerable anticancer activity *in vitro*, whereas its effect *in vivo* is limited. Here, potassium usnate (KU) was prepared by the salinization of UA to enhance its water solubility. KU showed increased bioavailability compared with UA in the tumor, liver, and plasma of a CT26 syngeneic mouse tumor xenograft model after oral administration, as determined by LC-MS/MS analysis. KU exhibited potent anticancer effects on colorectal cancer cells and inhibited liver metastasis in an orthotopic murine colorectal cancer model. KU treatment downregulated the epithelial-mesenchymal markers Twist, Snail, and Slug and the metastasis-related genes *CAPN1*, *CDC42*, *CFL1*, *IGF1*, *WASF1*, and *WASL* in cells and tumor tissues. The present results suggest the potential application of the water-soluble form of UA, KU, in anticancer therapy.

## Introduction

Drug oral bioavailability is positively related to reduced molecular flexibility and low polar surface area or total hydrogen bond count^1^. Excellent solubility and a satisfactory dissolution rate are essential conditions for the clinical application of candidate drugs.

Usnic acid (UA) is one of the most widely studied bioactive lichen secondary metabolites^2,3^. UA was first shown to have inhibitory activity against lung cancer cells^4,5^, and its effect on inhibiting proliferation was subsequently demonstrated in a wide variety of cancer cell lines^6^. Despite the promising anticancer activity of UA, it has not been developed for clinical application because of poor water solubility^7^ and high hepatotoxicity^8,9^. Therefore, the development of a bioavailable form of UA is an important issue in clinical research. Several strategies were proposed to improve the anticancer activity of UA *in vivo* by developing modified forms with high water solubility and potent antitumor activity. Studies focused on finding a suitable solvent for the solubilization of UA that showed no direct effects on any of the commonly used cell lines. 2-Hydroxypropyl-beta-cyclodextrin was identified as a solubilizing agent for UA that does not affect its antiproliferative activity against the human leukemia cell line K-562^10^. Nanoencapsulation was investigated as a method to improve the antitumor activity and reduce the hepatotoxicity of UA, and the encapsulation of UA into PLGA-nanocapsules produced a 26% increase in ascitic tumor formation (Sarcoma-180) in inoculated Swiss mice and a reduction in drug hepatotoxicity^11^. However, the underlying mechanism remains unclear. Also, Polyacrylamide complex formation with UA by establishment of strong acidic-base interactions made UA water soluble and showed enhanced antimicrobial activity^12^, and Nanocrystal suspensions of UA prepared by wet milling method showed higher bioavailability in rats^13^. Another study demonstrated that the cytotoxic activity of UA against L1210 cells can be improved by conjugation to a polyamine chain^14^. UA was derivatized with various amine moieties to improve drug cellular uptake by targeting the polyamine transport system (PTS). Although the results showed an increase in drug cytotoxicity against human cancer cell lines, drug targeting to the PTS was unsuccessful. In the present study, potassium usnate (KU) was generated using a previously described method, and its anticancer activity was examined *in vitro* and *in vivo* experimental model of colorectal cancer as colorectal cancer is one of the most common cancer types and, despite of advances in developing chemotherapy regimen, patients with the cancer suffer from local recurrence with chemoresistance and/or metastasis with 40–80% to liver^15^. The oral bioavailability of KU was enhanced significantly after oral administration in a CT26 syngeneic mouse xenograft model, as determined by LC-MS/MS detection. KU showed potent anticancer activity against colorectal cancer cell lines, and KU administration suppressed tumor growth in a mouse liver metastasis model. The findings of the present study suggest a promising solution for the low water solubility-related limitation to the use of UA in clinical anticancer therapy.

## Results

### UA shows anticancer activity in colorectal cancer cells with limited *in vivo* efficacy

**Figure 1:**
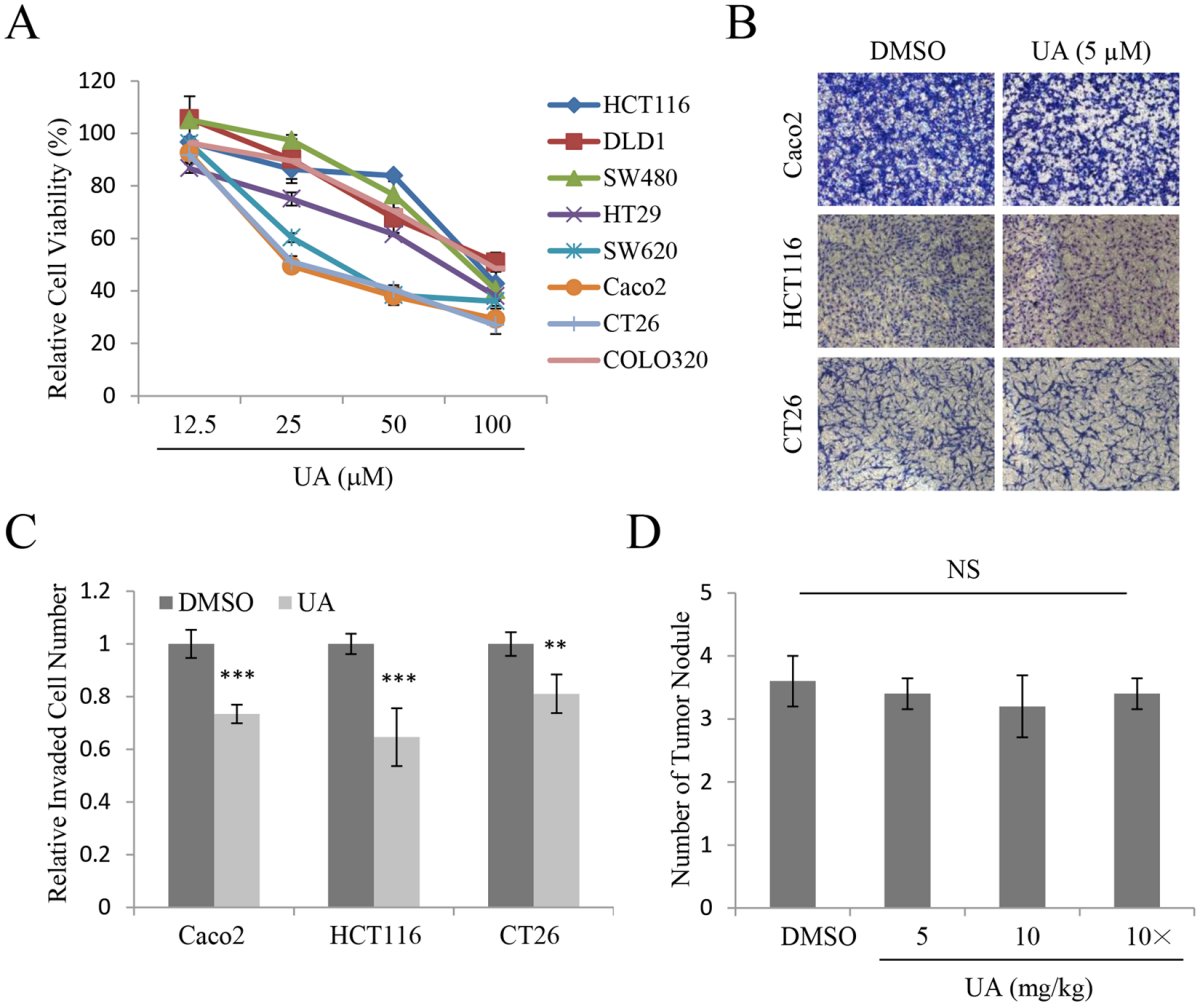
Usnic acid showed *in vitro* anticancer activity in colorectal cancer cells. (**A**) Relative cell viability of HCT116, DLD1, SW480, HT29, SW620, Caco2, CT26, and COLO320 cells treated with usnic acid (UA). (**B,C**) Invasion assays in Caco2, HCT116, and CT26 cells treated with 5 μM UA (**B**), and quantification of invaded cell numbers in each group (**C**). (**D**) Quantitative analysis of metastasis score in isolated mouse liver tissues from orthotopic liver metastasis models (n = 4 each group). “10x” denote that UA was administered ten times within 2 weeks, while without indication denotes that of six times. Results are reported as the mean ± standard error of the mean.

In a previous study, we demonstrated the cytotoxic, anti-motility, and anticancer activity of UA against several cancer cells^16,17^. Also, it was reported that UA has cytotoxic activity against HCT116, human colorectal cancer cells^18^. Here, to further examine the anticancer effect of UA on colorectal cancer, human colorectal cancer cell lines including HCT116, DLD1, SW480, HT29, SW620, Caco2, and COLO320 and the CT26 mouse colon carcinoma cell line were exposed to UA at concentrations of 12.5–100 μM, and cell viability was assessed with the MTT assay. As shown in Fig. 1A, UA showed cytotoxic activity in all the tested colorectal cancer cells. To further explore the anti-invasive activity of UA in colorectal cancer cells, Caco2, HCT116, and CT26 cells were subjected to invasion assays after exposure to 5 μM UA. The number of invaded cells was lower in the UA-treated groups than in the control groups (Fig. 1B). Quantitative analysis showed that the inhibitory effect of UA on the invasive ability of these colorectal cancer cells was significant (Fig. 1C). These results indicated that UA exhibits anticancer effects against human colorectal cancer cells.

The most deaths of colorectal cancer are associated with metastasis, and the liver, lung and peritoneum are common metastasis sites for colorectal cancer. Therefore, the *in vivo* efficacy of UA was then assessed in orthotopic liver metastasis mouse models. Firefly luciferase-expressing CT26 cells were inoculated via splenic injection to form multiple tumor foci in the livers of syngeneic BALB/c mice. On day 3 after tumor establishment, 5 or 10 mg/kg UA in DMSO in a total volume of 200 μL of PBS was administered via intraperitoneal injection (six or ten times within 2 weeks), and each mouse was analyzed by optical imaging on days 2, 9, and 16. Due to the solubility limit, the maximum dosage of UA is 10 mg/kg. Immediately after final imaging analysis, the liver and disseminated peritoneal tumors were excised and counted. Isolated mouse liver tissues from the control and treatment groups showed different numbers of tumor nodules of different sizes (Fig. S1). However, quantitative analysis of metastasis score did not detect a statistically significant difference in the metastasis score between the control and treatment groups (Fig. 1D). All animals in both control and treatment groups showed tumor progression as evidenced by increasing bioluminescence (data not shown). Taken together, these results indicated that UA had no significant inhibitory effect on metastasis in the *in vivo* orthotopic murine colorectal cancer model despite showing inhibitory activity against invasion in the *in vitro* experiments.

### KU shows enhanced oral bioavailability

**Figure 2:**
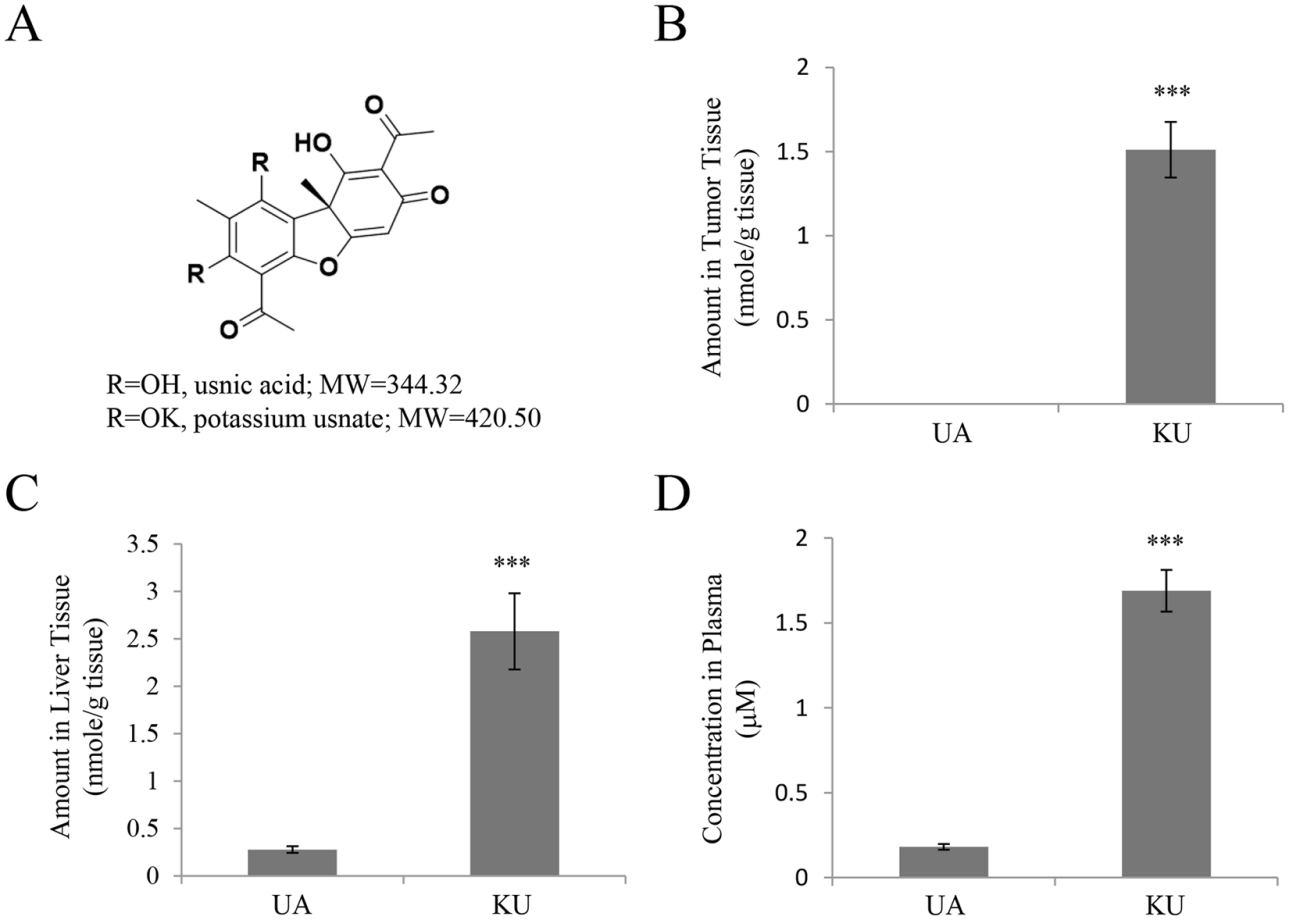
Usnate distribution in the tumor, liver, and plasma after oral administration of potassium usnate in a CT26 syngeneic mouse tumor xenograft model. (**A**) Chemical structure of UA and potassium usnate (KU). (**B–D**) Quantitative LC-MS/MS analysis of usnate in tumor tissues (**B**), liver tissues (**C**), and plasma (**D**) in a CT26 syngeneic mouse tumor xenograft model after oral administration of UA or KU. Results are reported as the mean ± standard error of the mean. ***P < 0.001.

As low solubility may account for the poor effect of UA *in vivo*, KU was synthesized by the salinization of UA; the water solubility of UA alone was measured as 52.2 ± 2.90 μM (17.7 ± 0.20 μg/mL) at 25 °C, pH 7.4 by μSol Method with DMSO stock solution; the structures and the physical properties of KU and UA are shown in Figs 2A and S2, respectively; the hydrolytic stability of UA was consistent at pH range of 4–7 in room temperature while KU was readily converted to its corresponding UA (Fig. S3). To determine whether the salt form of UA improved its bioavailability, the amount and concentration of usnate in the tumors, liver, and plasma of CT26 syngeneic tumor xenograft-bearing mice were measured by LC-MS/MS analysis. UA or KU was administered orally at a dose of 30 mg/kg at 7 days after CT26 cell inoculation, and the tumor tissues, liver tissues, and plasma of mice were collected after 16 h. As shown in Fig. 2B, usnate was undetectable in tumor tissues in mice receiving UA, whereas 1.5117 ± 0.166 nmol/g of tissue was detected in KU-treated mice. Assuming that the density of tumor tissue is 0.1 to 1.0 mL/g, the concentration of usnate in tumor tissue is about 1.51 to 15.1 μM. Similarly, the amounts of usnate detected in liver tissues were 0.2788 ± 0.034 nmol/g of tissue for UA and 2.5789 ± 0.402 nmol/g of tissue for KU administered mice (Fig. 2C); the plasma concentrations of usnate were 0.181 ± 0.016 μM for UA and 1.690 ± 0.122 μM for KU administered mice (Fig. 2D). Taken together, these results indicated that poor water solubility limited the effect of UA *in vivo*, and KU represents a potential form for *in vivo* administration as its oral bioavailability was higher than that of UA.

### KU shows potent *in vitro* anticancer activity in colorectal cancer cells

**Figure 3:**
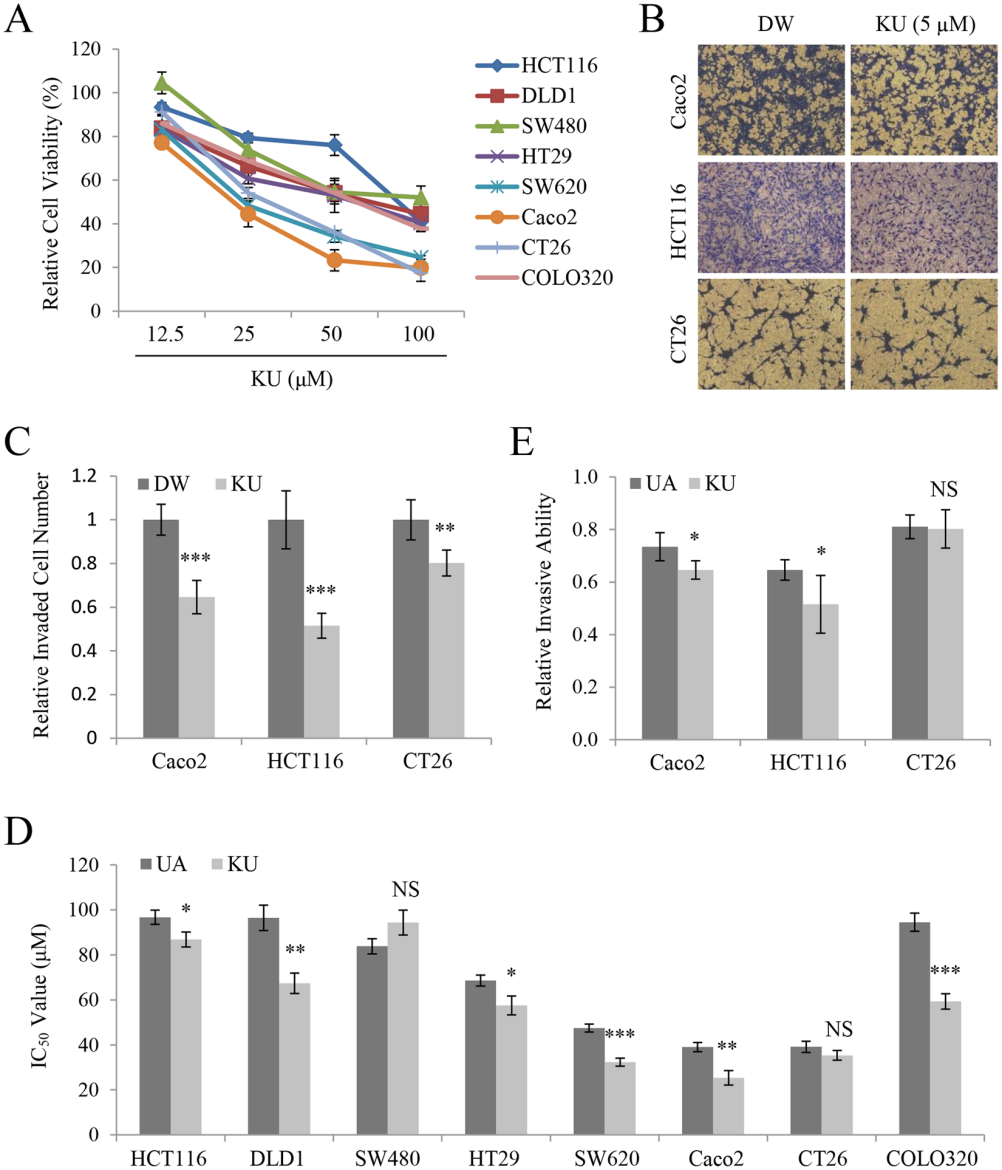
KU shows potent anticancer activity in colorectal cancer cells. (**A**) Relative viability of HCT116, DLD1, SW480, HT29, SW620, Caco2, CT26, and COLO320 cells treated with KU. (**B,C**) Invasion assays in Caco2, HCT116, and CT26 cells treated with 5 μM KU (**B**), and quantification of invaded cell numbers in each group (**C**). (**D**) Comparison of IC_50_ values between UA and KU. (**E**) Comparison of the relative invasive abilities of Caco2, HCT116, and CT26 cells treated with UA and KU. Results are reported as the mean ± standard error of the mean. *P < 0.05, **P < 0.01, ***P < 0.001.

To determine whether KU retained anticancer activity, MTT and invasion assays were performed in cells exposed to KU as described for UA. Similar to the results observed with UA, KU showed cytotoxic activity (Fig. 3A) and inhibited invasion (Fig. 3B,C) in colorectal cancer cells. To compare the effects of the two forms, IC_50_ values were calculated for UA and KU in the tested cells. The results showed a significantly lower IC_50_ value for KU than for UA except in SW480 and CT26 cells (Fig. 3D). At a non-cytotoxic concentration of 5 μM, KU showed more potent inhibitory activity against cell invasion than UA in Caco2 and HCT116 cells (Fig. 3E). These results suggested that KU retained the cytotoxicity and invasive inhibitory activity of UA.

### KU significantly decreases metastatic tumor nodule formation in the liver in the orthotopic murine colorectal cancer model

**Figure 4:**
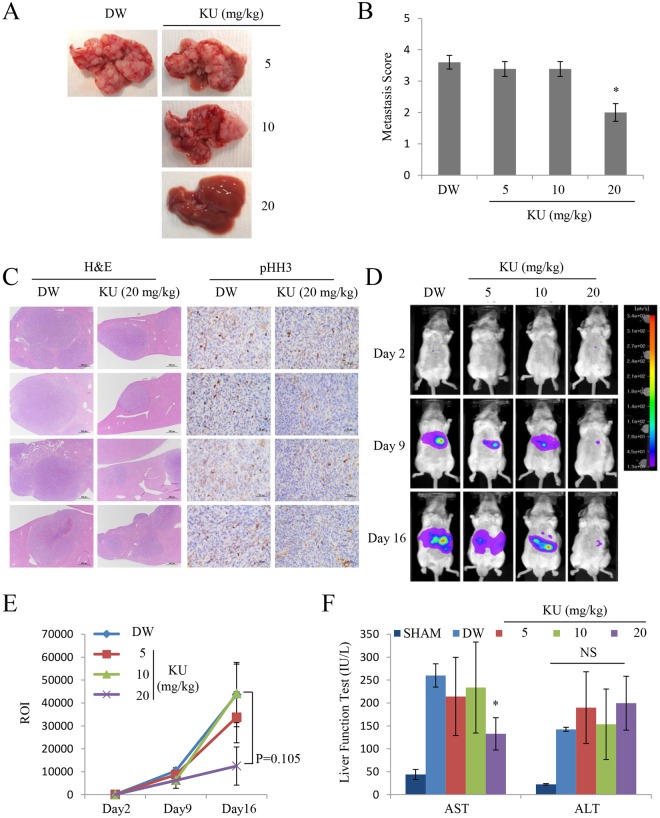
KU inhibits liver metastasis in an orthotopic murine colorectal cancer model. (**A**) Representative images of liver tissues isolated from four mice treated with DW or KU. (**B**) Quantitative analysis of metastasis score in isolated mouse liver tissues from the orthotopic liver metastasis model (n = 4 each group). (**C**) Hematoxylin and eosin staining and immunohistochemical analysis of phosphor-Histone H3 (pHH3) of isolated liver tissues from the mouse liver metastasis model. Scale bars, 500 μm. (**D**) Representative images of IVIS luciferase results in mice inoculated with colorectal cancer cells. (**E**) Quantitative analysis of signals from the IVIS luciferase images. On day 3 after tumor establishment, mice were analyzed by optical bioluminescence imaging at 2, 9, and 16 days after intraperitoneal KU administration (5, 10, and 20 mg/kg/mouse, three times a week). Control groups received DW instead of treatment. The average signal intensity of 20 mg/kg KU-treated mice was weaker than that of control mice (P = 0.105) at 16 days after tumor cell inoculation. (**F**) Liver function test results of AST and ALT levels in tumor tissue. Results are reported as the mean ± standard error of the mean, n = 4, *P < 0.05, **P < 0.01, ***P < 0.001.

A mouse liver metastasis model with firefly luciferase-expressing CT26 cells was established to determine the inhibitory activity of KU against metastasis *in vivo*. As described for UA, different doses of KU dissolved in DW (5, 10, and 20 mg/kg/mouse) in a total volume of 200 μL were administered via intraperitoneal injection (six times within 2 weeks). Immediately after the final imaging analysis, the liver and disseminated peritoneal tumors were excised and counted. The number of tumor nodules was significantly lower in the KU-treated group than in the control group as determined from isolated liver tissue images (Fig. 4A). Quantitative analysis and histologic examination showed that the metastasis score and tumor area were significantly lower in the 20 mg/kg KU-treated group than in the other groups (Figs 4B,C and S4). Also, immunohistochemical analysis revealed that nuclear staining of mitosis marker, phosphorylated histone H3 (pHH3)^19^, was lower in the 20 mg/kg KU-treated group than in the other groups (Fig. 4C). Optical imaging analysis on days 2, 9, and 16 showed tumor progression in all animals in both control and treatment groups, as evidenced by increasing bioluminescence (Fig. 4D). Quantitative analysis of the region of interest (ROI) showed that the bioluminescent signal was weaker in the 20 mg/kg KU-treated group than in the other groups (Fig. 4E, Table S1). Taken together, these results suggested that KU inhibited tumor growth in an orthotopic liver metastasis mouse model.

As UA causes hepatotoxicity and acute liver failure in patients receiving UA as an herbal supplement in weight loss agents^20^, the plasma levels of the liver functional enzymes aspartate aminotransferase (ASL) and alanine transaminase (ALT) were measured in the sham and liver metastasis mouse groups. These enzymes are released from liver cells when the liver is injured or damaged, and increased AST and ALT levels in the plasma indicate hepatic toxicity or disease. AST and ALT levels were higher in orthotopic liver metastasis mice than in the sham control group (Fig. 4F). In mice induced to form liver metastasis, an almost constant ALT level was maintained in both control and treatment groups, whereas AST levels were significantly decreased in mice injected with 20 mg/kg KU. As further severe liver damage was prevented by the therapeutic effects of KU on hepatic tumor growth, AST blood levels seemed to be decreased. These results indicated that KU at the doses administered had no hepatotoxic effects in a mouse liver metastasis model.

### KU downregulates epithelial-mesenchymal transition (EMT) markers and cell motility-related genes

**Figure 5:**
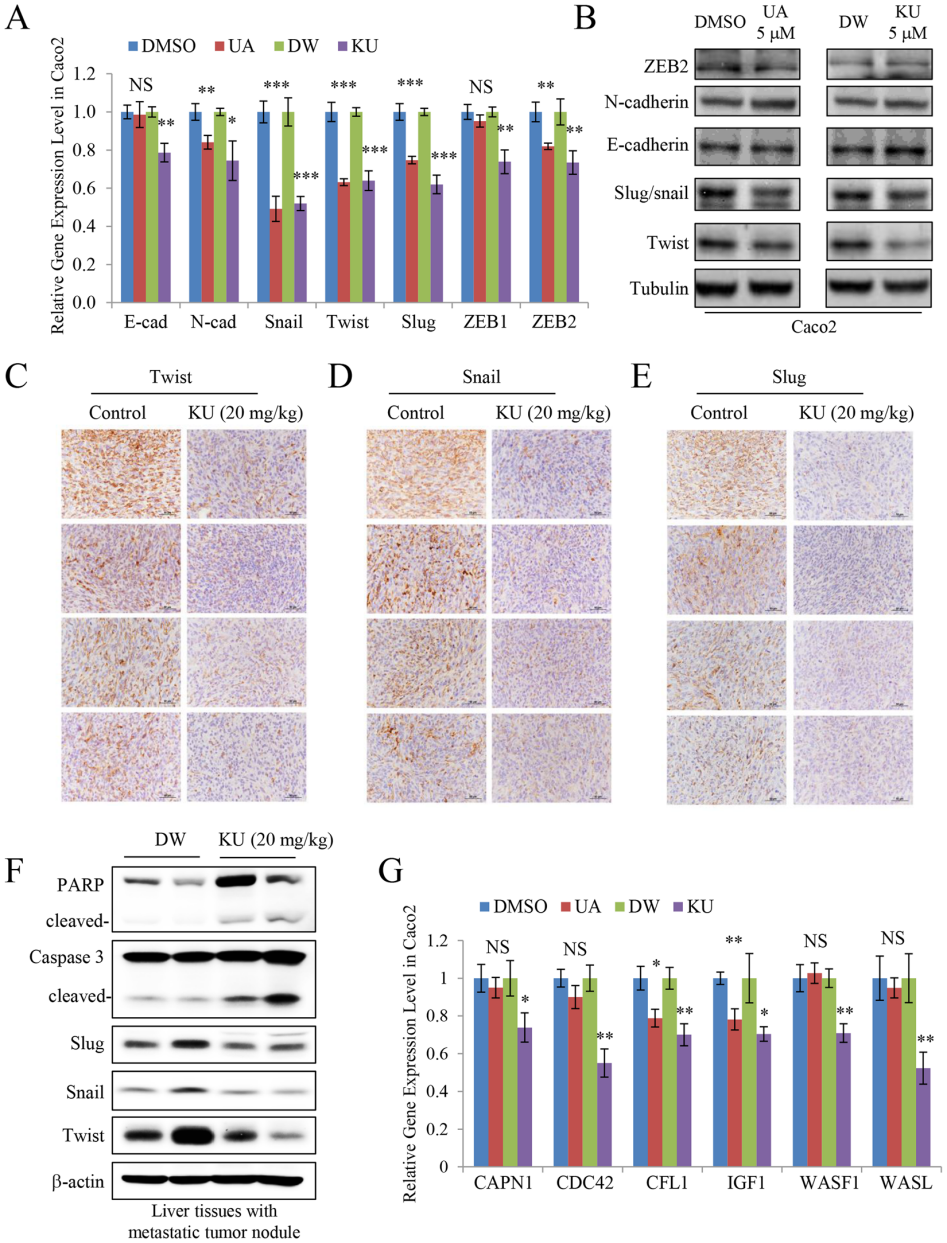
KU downregulates epithelial-mesenchymal transition markers and the expression of genes involved in cell motility. (**A**) Gene expression of EMT markers in Caco2 cells treated with UA and KU. (**B**) Protein levels of EMT markers in Caco2 cells treated with UA and KU. (**C–E**) Immunohistochemical analysis of Twist (**C**), Snail (**D**), and Slug (**E**) in isolated liver tissues from the mouse liver metastasis model administered with 20 mg/kg KU. Scale bar, 50 μm. (**F**) The levels of apoptosis and EMT markers in isolated liver tissues from the mouse liver metastasis model administered with 20 mg/kg KU (n = 2 each group). (**G**) mRNA expression levels of cell motility-related genes in Caco2 cells treated with UA and KU. Results are reported as the mean ± standard error of the mean. *P < 0.05, **P < 0.01, ***P < 0.001.

In our previous study, we showed that UA reduced the levels of epithelial-mesenchymal transition (EMT) markers in A549 lung cancer cells^16^. Changes in the expression and the levels of EMT markers were also observed in Caco2 cells treated with UA and KU. As shown in Fig. 5A, the mRNA levels of N-cadherin, Snail, Twist, Slug, and ZEB2 were significantly decreased by UA and KU treatment at 5 μM. However, at the protein level, only Twist, Snail, and Slug were downregulated in UA- and KU-treated cells (Fig. 5B). Consistently, isolated liver tissues from the mouse liver metastasis model administered with 20 mg/kg KU in Fig. 4 showed reduced levels of Twist, Snail, and Slug (Fig. 5C–F). In addition, increased cleavages of apoptosis markers were identified in the isolated liver tissues (Fig. 5F). Collectively, these results indicated that the antimetastatic and anticancer activity of KU may be attributed to the suppression of EMT in colorectal cancer cells.

The RT^2^ profiler^TM^ PCR assay was performed to screen for cell motility-related target genes of KU in Caco2 cells. The results showed that *CAPN1*, *CDC42*, *CFL1*, *IGF1*, *WASF1*, and *WASL* were significantly downregulated by KU treatment (Fig. 5G). However, only *CFL1* and *IGF1* gene expression levels were decreased by UA at the same concentration. These results provided a basis for future studies on the molecular mechanism underlying the KU-mediated anticancer activity.

## Discussion

The present study is the first to describe the cytotoxicity and inhibitory activity of UA in colorectal cancer cells. Water solubility can be considered as the main reason for the unsatisfactory performance of UA in *in vivo* experiments. Many strategies to increase the *in vivo* activity of UA have been investigated^10–15^. In this study, water-soluble potassium salts of UA were synthesized as previously described^21^. Obviously, high oral bioavailability is an important parameter for the selection of bioactive molecules as new drug candidates, and a deep understanding of the molecular properties that limit oral bioavailability can contribute to the design of new therapeutic agents. We found that the oral bioavailability of KU was significantly higher than that of UA in a CT26 inoculated xenograft mouse model, as determined by LC-MS/MS analysis of plasma and isolated tumor and liver tissues. These results suggested that water solubility was a limiting factor regarding the oral bioavailability of these molecules, and KU showed a high potential as a drug candidate.

KU retained the anticancer activity of UA in colorectal cancer cell lines *in vitro*, and the cytotoxicity and invasive inhibitory activity of KU were more potent than those of UA. Furthermore, KU suppressed tumor growth in our mouse liver metastasis model. KU was expected to show similar *in vitro* activity with UA when tested at low concentrations where the compound is thought to be completely dissolved. The potent anticancer activity of KU than UA can be explained possibly due to difference in reaching into intracellular action site. However, it is speculated that the intrinsic anticancer activity of usnate is unchanged by salinization of UA. As UA causes hepatotoxicity and acute liver failure when administered as an herbal supplement in weight loss agents^20^, KU-treated mice were subjected to liver function tests. As shown in Fig. 4F, the levels of the liver functional enzymes AST and ALT did not change significantly in the liver metastasis mouse groups, and the levels of AST were lower in mice treated with 20 mg/kg KU than in the control groups. These results suggested that KU does not cause additional hepatotoxicity when used as an anticancer drug, and the therapeutic effect of KU may prevent liver damage in some cases.

In previous work from our group, we demonstrated the anticancer activity of *F. cucullata* and UA against several cancer cell lines^17^. UA exhibits selective cytotoxicity in cancer cells by inducing apoptosis at lethal concentrations, and it inhibits tumorigenesis and the motility of cancer cells at sub-lethal concentrations. The effect of UA on metastasis inhibition was shown to involve the process of EMT. Here, we tested the expression of EMT-related molecules in response to UA and KU treatment. We found that UA or KU decreased the levels of Snail, Slug, and Twist at the mRNA and protein levels. Immunohistochemical analysis of Snail, Slug, and Twist in isolated liver tissues in the metastasis mouse model revealed consistent results, suggesting that the EMT signaling pathway was involved in the inhibitory activity of KU in colorectal cancer cells. In addition, RT^2^ profiler^TM^ screening identified cell motility target genes, such as *CAPN1*, *CDC42*, *CFL1*, *IGF1*, *WASF1*, and *WASL*, providing valuable information for future molecular mechanistic studies. The results of the present study suggested the potential application of KU in clinical cancer therapy.

In summary, UA showed anticancer activity in human colorectal cancer cell lines *in vitro*. To improve the *in vivo* bioactivity of UA for clinical application, KU was synthesized to increase the water solubility of the compound. The improved oral bioavailability demonstrated the potential of KU as a new drug candidate. In addition, the antitumor effect of KU in an orthotopic liver metastasis model suggested its potential as a clinical anticancer drug. However, further studies are necessary to elucidate the molecular mechanisms underlying the metastasis inhibitory activity of KU.

## Materials and Methods

### Preparation of KU

KU was prepared following a previously published method^10^. Briefly, 200 mg of UA (Sigma, St. Louis, MO, USA) was partially dissolved in 40 mL of water at 40 °C and then mixed with 10% KOH until the compound was completely solubilized. The solution was frozen at −80 °C and lyophilized, and the dried powder was dissolved in autoclaved distilled water (DW) before use. The structure of KU was confirmed by nuclear magnetic resonance.

### Cell culture

The human colorectal cancer cell lines HCT116, DLD1, SW480, HT29, SW620, Caco2, and COLO320, and the mouse colorectal cancer cell line CT26, were used in this study^22^. Cells were cultured in DMEM supplemented with 10% fetal bovine serum and 1% Penicillin-Streptomycin solution under a humidified 5% CO_2_ atmosphere at 37 °C in an incubator.

### Cell viability assay

Colorectal cancer cell growth and survival were detected with the colorimetric quantification of 3-(4,5-dimethylthiazol-2-yl)-2,5-diphenyltetrazolium bromide using the MTT assay^23^. Briefly, cells were seeded and cultured in 96-well plates (2.5 × 10^3^ cells/well), and treated with UA or KU for 48 h. After DMSO dissolution, the absorbance at 540 nm was determined using a microplate reader with Gen 5 (2.03.1) software (BioTek Eon, VT, USA).

### Cell invasion assay

Invasion assays were performed in Boyden chambers (Corning, New York, USA)^16^. A 120 μL Cells (2 × 10^6^) suspended in medium (DMEM containing 0.2% BSA dissolved in PBS) treated with or without UA or KU were seeded in the upper transwell chamber (Corning, New York, USA) coated with gelatin. Then, fibronectin (10 μg/mL) was added into the lower chamber medium (400 μL) as a chemotactic agent.Then invaded cells adhering to the underneath of upper chamber were fixed and stained with the Diff Quik kit (Sysmex) after 24 h of incubation. The quantitation of the cells were performed under an upright microscope (5 fields/chamber). Each invasion assay was repeated in three independent experiments.

### Orthotopic liver metastasis models

Five-week-old male BALB/c mice with pathogen-free condition were obtained from Orient Company (Seongnam, Korea). According to the Guiding Principles in the Care and Use of Animals (DHEW publication, NIH 80–23), the handling of animals and all *in vivo* experiments were performed. The Chonnam National University Medical School Research Institutional Animal Care and Use Committee approved the experimental protocol.

A syngeneic mouse model of colorectal cancer metastasis to the liver was established by infusion of tumor cells into the portal system via intrasplenic injection^24^. Briefly, a left subcostal incision was performed under isoflurane anesthesia, and CT26 cells expressing firefly luciferase (CT26-Fluc) (1 × 10^5^ cells) in 50 μL of PBS were inoculated by intrasplenic injection. For optical live imaging in this model, a splenectomy was performed immediately after inoculation of tumor cell, and the firefly luciferase activity was analyzed at several time points with the Xenogen system. After imaging analysis, the liver and disseminated peritoneal tumors were excised and weighed. Weight of tumor was determined by subtracting the average weight of liver of normal mice (n = 4) from the weight of total tumor-bearing liver with addition of the weight of total extrahepatic tumors. UA or KU (5, 10, or 20 mg/kg/mouse) was given via intraperitoneal injection 3 days after inoculation of tumor cell. To evaluate inhibition of tumor growth, mice were analyzed every 7 days with the use of optical imaging. On day 21 after inoculation of tumor, mice were sacrificed and a laparotomy was performed. Metastatic tumor nodules in the liver with a diameter of >1.0 mm were counted using a microscope, and a metastasis score was assigned based on nodule size as follows: 0 (no gross metastasis), 1 (tumor size >1 mm), and 10 (tumor size >5 mm). The metastasis score was multiplied by the number and the score of nodules.

### Optical imaging analysis

Examination of bioluminescence in cultured cells or live mice was performed by optical imaging using a cooled CCD system (Xenogen IVIS). Photographic images of the tissues with gray-scale background were overlaid with color images of bioluminescent signals using Living Image and IGOR-PRO image analysis software (Wave Metrics). For *in vitro* imaging, CT26-Fluc cells subjected to lentiviral vector transduction were confirmed with the Xenogen system at 2 min after addition of D-luciferin. For *in vivo* imaging, anesthetization of mice were indueded with ketamine (100 mg/kg) and xylazine (10 mg/kg), and 2 min after intraperitoneal (ip) administration with D-luciferin (2 mg/mouse), analysis of bioluminescent signals were performed with the Xenogen system with a 1 min acquisition time.

### Sample preparation for LC-MS/MS analysis

Stock solutions (10 mM) of UA or KU were prepared by dissolution in DMSO. Stock standard solutions were stored at −20 °C. A working standard solution was prepared by dilution of the stock standard solution with a mixture of acetonitrile-water (1:1, v/v). The working standard solution was serially diluted to prepare a concentration series of 0.975, 3.9, 15.6, 62.5, 250, 1000, and 4000 nM in 50% acetonitrile.

Skin tumor xenografts were established using CT26 cells^25^. After 7 days of inoculation and gross tumor formation, UA or KU at 30 mg/kg was administered orally, and tumor, liver, and plasma samples were collected after 16 h (n = 9 each). Mouse tumor and liver samples were homogenized in four volumes of ice-cold deionized water. Plasma and homogenized tissue samples were prepared for analysis in a 96-well cluster tube plate by protein precipitation. A volume of 30 µL of each sample was transferred to eight-well tube strips placed in an 8 × 12 rack (VWR, Emeryville, CA, USA). Four volumes of ice-cold extraction solution (acetonitrile) containing 4-methylumbelliferone (4-MUF, internal standard) were added and vortexed for 10 min. After sonication for 30 min, samples were placed on ice for 1 h. The 96-well cluster tube plate was centrifuged at 1000 × *g* for 10 min, and the supernatant was analyzed by LC-MS/MS. Standard samples were prepared in the same manner using a blank matrix.

### LC-MS/MS analysis

Samples were analyzed with the Prominence UPLC system (Shimadzu, Japan) equipped with an API 3200 QTRAP™ LC-MS/MS system (Applied Biosystems, Foster City, CA, USA). Sample volumes of 10 μL were injected into an Aquity HSS UPLC C_18_ column (2.5 × 100 mm, 1.8 µm i.d.; Waters, Milford, MA, USA) and maintained at 30 °C. The column was pre-equilibrated in 100% v/v solvent A (deionized water containing 0.1% v/v formic acid)/0% v/v solvent B (acetonitrile containing 0.1% v/v formic acid) at a flow rate of 0.3 mL/min. The optimized LC elution conditions were 0.0–1.0 min, 0% B; 1.1–2.0 min, 50% B; 2.1–6.0 min, 98% B; 6.0–6.01 min, 0% B; and 6.01–8.0 min, 0% B. The overall chromatographic run time was 8 min. The autosampler compartment was maintained at 10 °C throughout the analysis. The retention times of UA, KU, and IS (4-MUF) were 4.52, 4.52, and 3.76 min, respectively. The ESI source was operated at −4500 V and 500 °C in a negative mode. Quadrupoles Q1 and Q3 were set on unity resolution. The samples were analyzed via multiple reaction monitoring. The monitoring ions were set as m/z 343 → 839 for UA and m/z 175 → 133 for 4-MUF. The acquisition and analysis of data were performed with Analyst™ software (version 1.5.2; Applied Biosystems).

### Histological examination and immunohistochemistry

Resected livers were fixed in 10% neutral-buffered formalin for 3 days. Then, the organs were dissected, embedded in paraffin, and stained with hematoxylin for histopathological evaluation. Tissue sections were immunostained with specific antibodies against phospho-Histone H3 (1:9000, code: 9701, Cell Signaling, Danvers, MA, USA), Slug (dilution: 1:400, code: 9585, Cell Signaling), Snail (dilution: 1:100, code: sc-28199, Santa Cruz, Dallas, TX, USA), and Twist (dilution: 1:100, code: ab50887, Abcam, Cambridge, UK) using a Bond-max system (Leica Microsystems, Bannockburn, IL, USA). Programmed heat-induced epitope retrieval was performed for 15 min using citrate-based pH 6.0 BOND epitope retrieval solution 1 (Slug) or EDTA-based pH 9.0 epitope retrieval solution 2 (Snail and Twist).

### Western blotting

Cells treated with UA or KU for 24 h were washed twice with ice-cold PBS and lysed in lysisbuffer^26^. Antibodies against E-cadherin (61018, BD Biosciences, San Diego, CA, USA), N-cadherin (610921, BD Biosciences), Snail/Slug (ab180714, Abcam, Cambridge, MA, USA), Twist (ab49254, Abcam), PARP (9542, Cell Signaling), Caspase-3 (9662, Cell Signaling), α-Tubulin (2125, Cell Signaling), and ZEB2 (HPA003456, Sigma) were detected with horseradish peroxidase-conjugated secondary antibody (Thermo Fisher Scientific, Waltham, MA, USA) with the use of Immobilon Western Chemiluminescent HRP Substrate Kit (Merck Millipore, Billerica, MA, USA) and luminescence imaging (Image Quant LAS 4000 mini). Multi-Gauge 3.0 was used to measure bands, and relative density was calculated based on the density of the α-tubulin bands in each sample. Expression of values were as arbitrary densitometric units corresponding to signal intensity.

### Gene expression analysis by PCR

The RT² Profiler™ PCR Array (330231, SA Biosciences, Qiagen, Courtaboeuf, France) was used to examine the expression patterns of 84 genes involved in human cell motility as previouslydescribed^27^. The manufacturer’s instructions were strictly followed. Gene expression levels were analyzed using the web-based software ‘RT2 Profiler PCR Array Data Analysis version 3.5′. Six cell motility-related genes, including *CAPN1*, *CDC42*, *CFL1*, *IGF1*, *WASF1*, and *WASL*, were selected as candidates.

### Quantitative RT-PCR (qRT-PCR)

Quantitative RT-PCR (qRT-PCR) was performed as previously described^23^. Briefly, total RNA (1 mg) isolated from UA- or KU-treated Caco2 cells using RNAiso Plus (TaKaRa, Otsu, Shiga 520–2193, Japan) was used to synthesize cDNA using a M-MLV reverse transcriptase kit (Invitrogen, Carlsbad, CA, USA) and SYBR green (Enzynomics, Seoul, Korea). The primers used for real-time PCR were as follows: E-cadherin (forward) 5′-cagaaagttttccaccaaag-3′ and (reverse) 5′-aaatgtgagcaattctgctt-3′; N-cadherin (forward) 5′-ctcctatgagtggaacaggaacg-3′ and (reverse) 5′-ttggatcaatgtcataatcaagtgctgta-3′; Snail (forward) 5′-tcccgggcaatttaacaatg-3′ and (reverse) 5′-tgggagacacatcggtcga-3′; Twist (forward) 5′-cgggagtccgcagtctta-3′ and (reverse) 5′-tgaatcttgctcagcttgtc-3′; Slug (forward) 5′-cgaactggacacacatacagtg-3′ and (reverse) 5′-ctgaggatctctggttgtggt-3′; ZEB1(forward) 5′-atgacacaggaaaggaagg-3′ and (reverse) 5′-agcagtgtcttgttgtag-3′; ZEB2 (forward) 5′-caagaggcgcaaacaagcc-3′ and (reverse) 5′-ggttggcaataccgtcatcc-3′; CAPN1 (forward) 5′-cctgcttgagaaggcctatg-3′ and (reverse) 5′-ggtccacgttgttccactct-3′; CDC42 (forward) 5′-aggctctctagtttaataaaaatcatgg-3′ and (reverse) 5′-gtttgtttaatacatctgaaaagaatgc-3′; CFL1 (forward) 5′-caaggatgccatcaagaa-3′ and (reverse) 5′-atccttagcctcctcgta-3′; IGF1 (forward) 5′-gatacacatcatgtcgtcttcaca-3′ and (reverse) 5′-cagtacatctccagtctcctcaga-3′; WASF1 (forward) 5′-tcctgatgttttaaaagaagaaacact-3′ and (reverse) 5′-aaaagtttttaactcctataggcaagc-3′; WASL (forward) 5′-agtggaggtctctgattggcc-3′ and (reverse) 5′-tctcctttcagggtctccca-3′; and GAPDH (forward) 5′-atcaccatcttccaggagcga-3′ and (reverse) 5′-agttgtcatggatgaccttggc-3′. qRT-PCR reactions and analyses were performed using CFX (Bio-Rad, Hercules, CA, USA).

### Statistical analysis

Data are presented as the mean ± standard error of the mean obtained from three independent experiments unless otherwise indicated. The Student’s *t*-test was utilized to determine statistical significance between two groups, and analysis of variance was utilized between three or more groups, respectively. *P*-values of < 0.05 are considered statistically significant.

## Electronic supplementary material

Dataset 1
