# Supplementary material for: Potassium usnate, a water-soluble usnic acid salt, shows enhanced bioavailability and inhibits invasion and metastasis in colorectal cancer
Source: Sci Rep. 2018 Nov 2;8:16234. doi: 10.1038/s41598-018-34709-9 (PMC6214985; doi:10.1038/s41598-018-34709-9)
Supplement: Supplementary file 1 — Dataset 1 [file 41598_2018_34709_MOESM1_ESM.doc]

**Supplementary Data for**

Potassium usnate, a water-soluble usnic acid salt, shows enhanced bioavailability and inhibits invasion and metastasis in colorectal cancer

Yi Yang ^a, b, 1^, Woo Kyun Bae ^c, 1^, Ji-Yoon Lee ^d^, Yong Jae Choi ^d^, Kyung Hwa Lee ^e^, Myong-Suk Park ^c^, Young Hyun Yu ^a^, So-Yeon Park ^a^, Rui Zhou ^a^, İsa Taş ^a, b^, Chathurika Gamage ^a, b^, Man-Jeong Paik ^a^, Jae Hyuk Lee ^e^, Ik Joo Chung ^c^, Kyung Keun Kim ^f^, Jae-Seoun Hur ^b^, Sang Kyum Kim ^d^, Hyung-Ho Ha ^a,*^, and Hangun Kim ^a,*^

^a^College of Pharmacy and Research Institute of Life and Pharmaceutical Sciences, Sunchon National University, Sunchon, Korea; ^b^Korean Lichen Research Institute, Sunchon National University, Sunchon, Korea; ^c^Department of Hematology-Oncology, Chonnam National University Medical School, Gwangju, Korea; ^d^College of Pharmacy, Chungnam National University, Daejeon, Korea; ^e^Department of Pathology, Chonnam National University Medical School, Gwangju, Korea; ^f^Department of Pharmacology, Chonnam National University Medical School, Gwangju, Korea

^1^These authors contributed equally to this work

Supplementary Data

**Supplementary Table 1.** The individual values of bioluminescent signals in region of interest (ROI) from IVIS luciferase images shown in Figure 4E.

**Supplementary Figure 1.** Representative images of liver tissues isolated from four mice treated with DMSO or UA.

**Supplementary Figure 2. Physical properties of UA and KU. (A) Color of KU. (B) Yield of reaction of the KU. (C) Melting point of UA. (D) Melting point of KU.** (E) ^1^H-NMR spectrum of UA. (F) ^1^H-NMR spectrum of KU. (G) MS spectrum of UA&KU.

**Supplementary Figure 3.** **The stability of UA at the pH range of 4–7 in room temperature. (A) UA stability at pH 7.0. (B) UA stability at pH 6.8. (A) UA stability at pH 6.0. (A) UA stability at pH 4.5**

**Supplementary Figure 4.** Quantitation of hematoxylin and eosin stained tumor area in isolated liver tissues from the mouse liver metastasis model shown in Figure 4C. (A) The individual hematoxylin and eosin stained tumor areas in isolated liver tissues. (B) Quantitation of hematoxylin and eosin stained tumor area in isolated liver tissues

**Supplementary Figure 5.** Full-length blots shown in Figure 5B.

**Supplementary Figure 6.** Full-length blots shown in Figure 5F.

Supplementary Table 1.


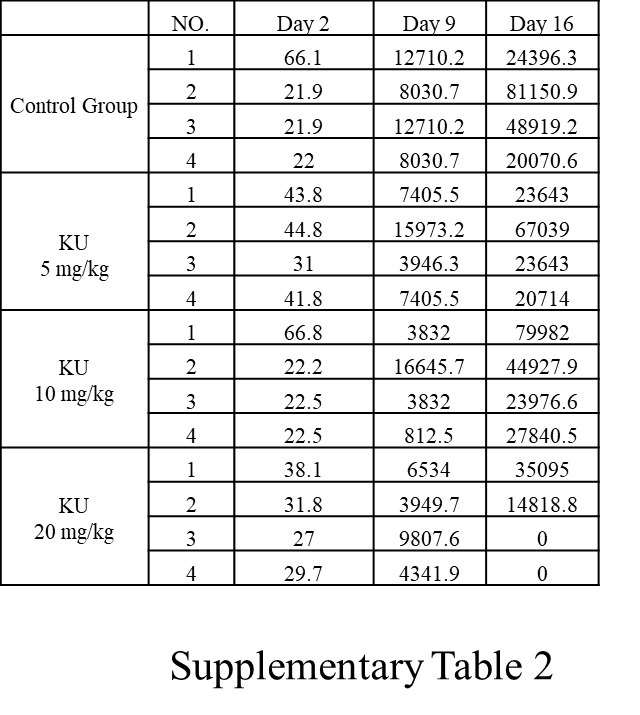


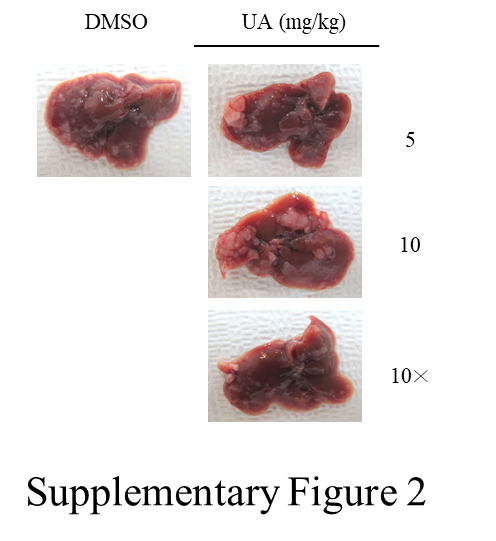


Supplementary Figure 1

**Supplementary Figure 2**

(A) Color of KU = Brown solid

(B) Yield of reaction of the KU = 92%

(C) Melting point of UA = 205.21^o^C

(D) Melting point of KU = 244.18^o^C (Melting point is unstable as KU is hydroscopic)

(E) ^1^H-NMR spectrum of UA


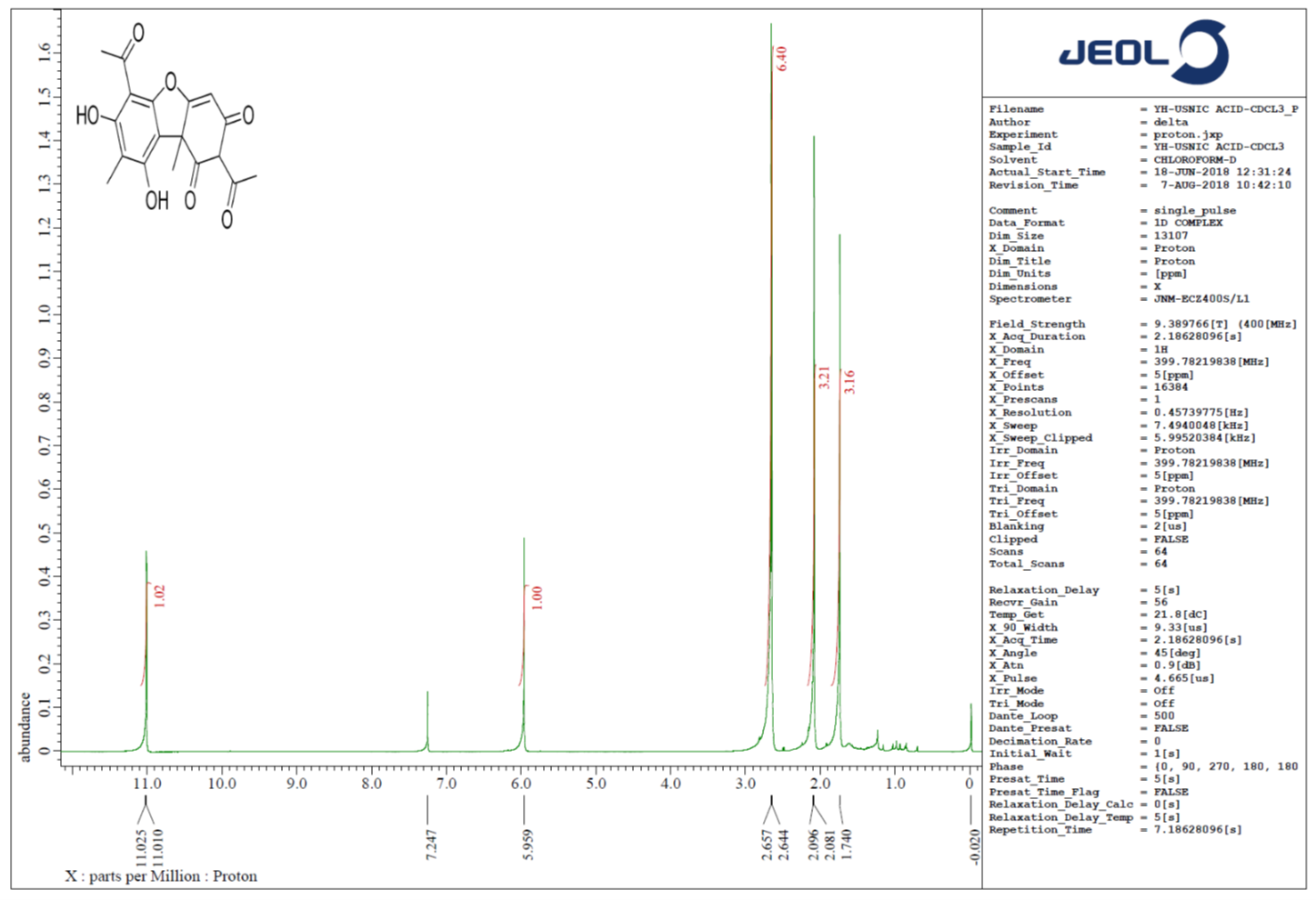


**UA ^1^H NMR;** ^1^H-NMR (400 MHz, CHLOROFORM-D) δ 11.02 (d, J = 5.9 Hz, 1H), 5.97 (d, J = 5.9 Hz, 1H), 2.65 (d, J = 5.0 Hz, 6H), 2.09 (d, J = 5.9 Hz, 3H), 1.74 (s, 3H)

(F) ^1^H-NMR spectrum of KU


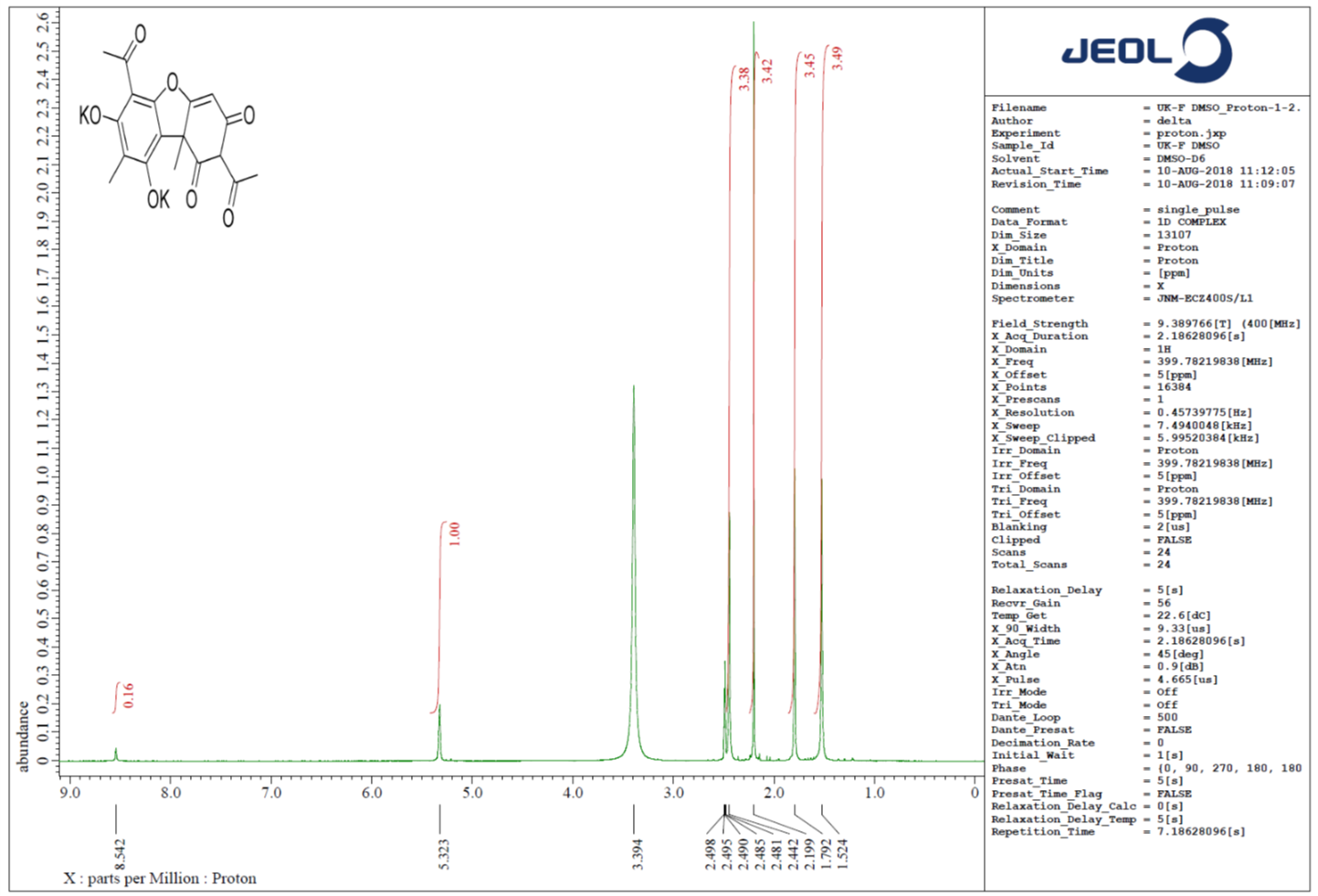


**KU 1H NMR;** ^1^H-NMR (400 MHz, DMSO-D6) δ 8.54 (s, 0H), 5.32 (s, 1H), 2.44 (s, 3H), 2.20 (s, 3H), 1.79 (s, 3H), 1.52 (s, 3H)

(G) MS spectrum of UA&KU = Positive[344.6], Negative[343.1]

Supplementary Figure 3

(A) UA stability at pH 7.0 (B) UA stability at pH 6.8

(C) UA stability at pH 6.0 (D) UA stability at pH 4.5


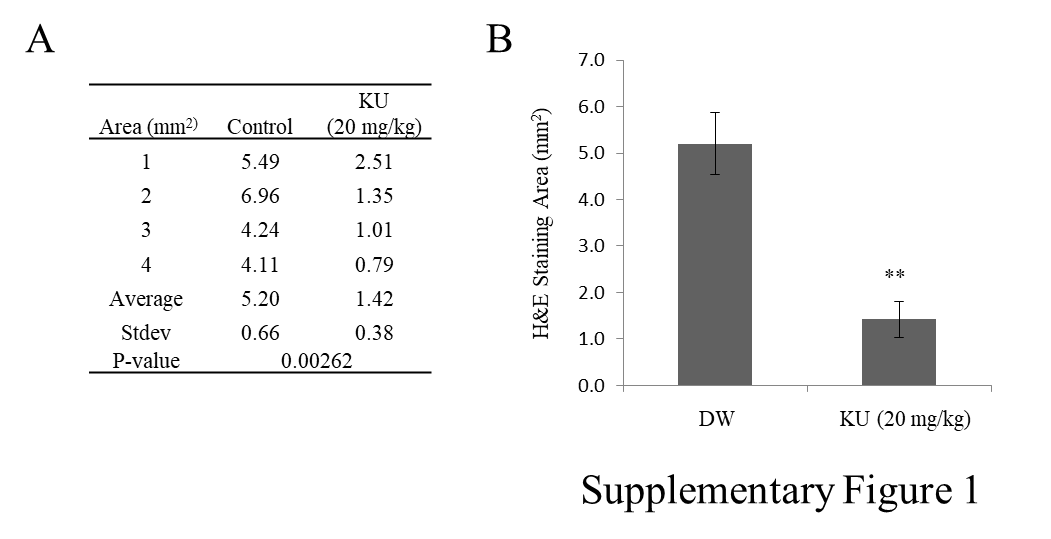


Supplementary Figure 4


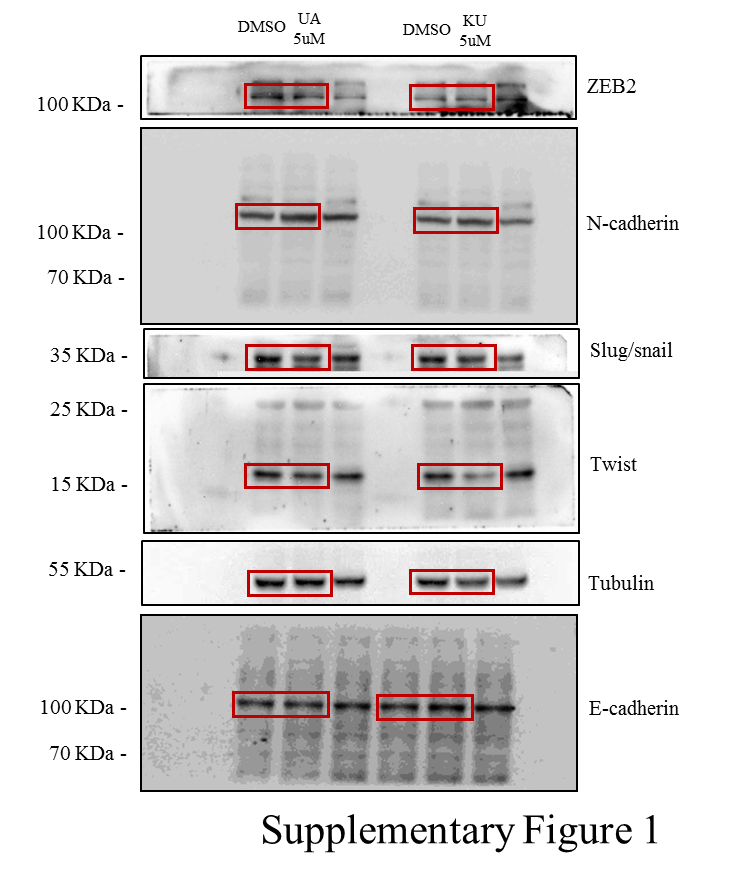


Supplementary Figure 5


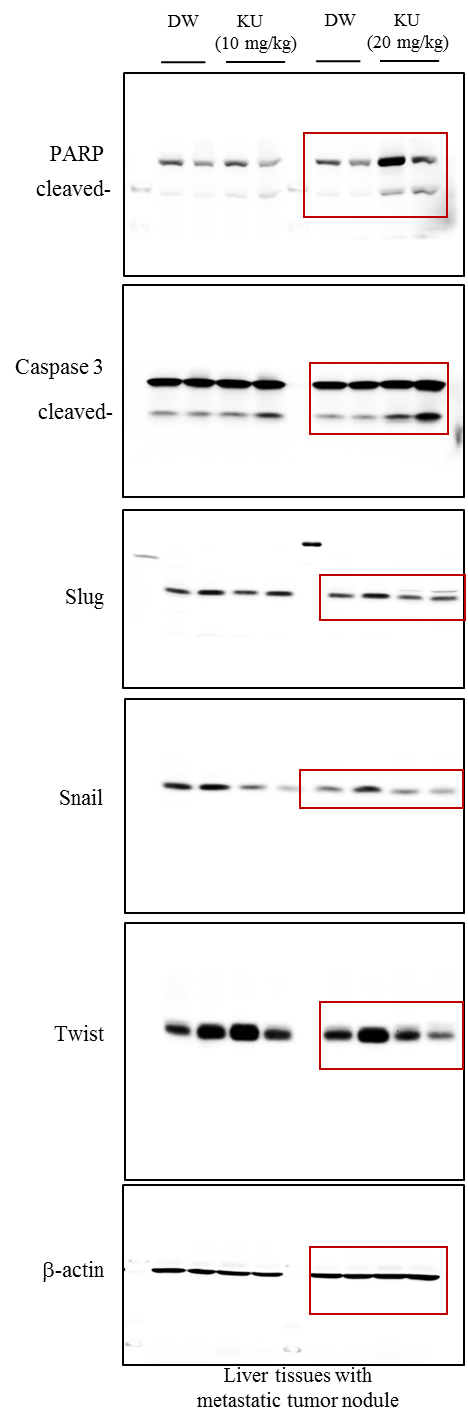


Supplementary Figure 6
